# The Regulation of Alfalfa Saponin Extract on Key Genes Involved in Hepatic Cholesterol Metabolism in Hyperlipidemic Rats

**DOI:** 10.1371/journal.pone.0088282

**Published:** 2014-02-05

**Authors:** Yinghua Shi, Rui Guo, Xianke Wang, Dedi Yuan, Senhao Zhang, Jie Wang, Xuebing Yan, Chengzhang Wang

**Affiliations:** College of Animal Science and Veterinary Medicine, Henan Agricultural University, Zhengzhou, Henan, China; Institut d'Investigacions Biomèdiques August Pi i Sunyer, Spain

## Abstract

To investigate the cholesterol-lowering effects of alfalfa saponin extract (ASE) and its regulation mechanism on some key genes involved in cholesterol metabolism, 40 healthy 7 weeks old male Sprague Dawley (SD) rats were randomly divided into four groups with 10 rats in each group: control group, hyperlipidemic group, ASE treatment group, ASE prevention group. The body weight gain, relative liver weight and serum lipid 1evels of rats were determined. Total cholesterol (TC) and total bile acids (TBA) levels in liver and feces were also measured. Furthermore, the activity and mRNA expressions of *Hmgcr, Acat2, Cyp7a1* and *Ldlr* were investigated. The results showed the following: (1) The abnormal serum lipid levels in hyperlipidemic rats were ameliorated by ASE administration (both ASE prevention group and treatment group) (*P*<0.05). (2) Both ASE administration to hyperlipidemic rats significantly reduced liver TC and increased liver TBA level (*P*<0.05). TC and TBA levels in feces of hyperlipidemic rats were remarkably elevated by both ASE administration (*P*<0.05). (3) mRNA expressions of *Hmgcr* and *Acat2* in the liver of hyperlipidemic rats were remarkably down-regulated (*P*<0.05), as well as mRNA expressions of *Cyp7a1* and *Ldlr* were dramatically up-regulated by both ASE administration (*P*<0.05). The activities of these enzymes also paralleled the observed changes in mRNA levels. (4) There was no significant difference between ASE treatment and ASE prevention group for most parameters evaluated. Our present study indicated that ASE had cholesterol-lowering effects. The possible mechanism could be attributed to (1) the down-regulation of *Hmgcr* and *Acat2*, as well as up-regulation of *Cyp7a1* and *Ldlr* in the liver of hyperlipidemic rats, which was involved in cholesterol biosynthesis, uptake, and efflux pathway; (2) the increase in excretion of cholesterol. The findings in our study suggested ASE had great potential usefulness as a natural agent for treating hyperlipidemia.

## Introduction

Hyperlipidemia is common in the worldwide population, and is considered as a highly modifiable risk factor for cardiovascular disease such as coronary heart disease and peripheral artery diseases. Elevated blood lipid levels, especially increased serum low-density lipoprotein (LDL) level, can accelerate atherosclerosis. Therefore, reducing high lipid levels has been regarded to be an important approach to prevent or slow the progression of atherosclerosis [1]. In recent decades, increasing attention has been paid to new natural agents with lipid-reducing activity [2]–[4]. The cholesterol-lowering effects and prevention in cardiovascular disease of saponins have been demonstrated [5], [6]. Alfalfa (*Medicago sativa*) is used as a food additive in the USA, Russia, China and North Africa, for its high contents of vitamin and bioactive ingredients [7], [8]. Alfalfa saponins (AS) are naturally bioactive compounds, which consist of a sugar moiety glycosidically linked to a hydrophobic aglycone (sapogenin), mainly pentacyclic triterpenoid [9]. AS has been found to reduce serum or plasma cholesterol in several species of experimental animals and evidence indicates that these changes result from a decreased cholesterol absorption and/or an increase in fecal steroid excretion [10]–[14]. However, no research has been carried out concerning whether AS can regulate the expression of some key genes involved in cholesterol metabolism, and whether the cholesterol-lowering effects of AS are mediated by these genes.

Cholesterol homeostasis is tightly controlled by coordinated changes in the concentrations of mRNA encoding multiple enzymes involved in cholesterol biosynthesis, uptake, and efflux pathway [15]. 3-Hydroxy-3-methylglutaryl CoA reductase (HMGCR) is the rate-limiting enzyme in cholesterol biosynthesis [16]. Cytochrome P450, family 7, subfamily a, polypeptide 1, also known as Cholesterol 7-alpha-hydroxylase (CYP7A1) is the rate-limiting enzyme in bile acid biosynthesis, a major efflux pathway for the elimination of cholesterol from the body [17]. Acyl-CoA: cholesterol O-acyltransferase 2 (ACAT2), is the major tissue cholesterol-esterifying enzyme [18]. LDL receptor (LDLR) plays a vital role in the hepatic uptake and clearance of plasma cholesterol [19]. Alfalfa saponin extract (ASE) was extracted from the leaves and stem of alfalfa. Our previous study have shown that ASE could decrease serum levels of total cholesterol (TC), triglycerides (TG) and low-density lipoprotein cholesterol (LDL-C) in piglets and rats [20], [21], but the regulation mechanism of ASE on cholesterol metabolism is still not understood. Based on the animal studies, it is important to ascertain a mechanism for the action of ASE on cholesterol metabolism. This study was therefore undertaken to investigate the cholesterol-lowering effects of ASE and its regulation mechanism on key genes implicated in cholesterol metabolism, i.e. 3-Hydroxy-3-methylglutaryl CoA reductase (*Hmgcr*), acyl-CoA: cholesterol O-acyltransferase 2 (*Acat2*), cytochrome P450, family 7, subfamily a, polypeptide 1 (*Cyp7a1*) and low-density lipoprotein receptor (*Ldlr*).

## Materials and Methods

### Animals

The total of 40 adult male healthy Sprague Dawley (SD) rats at the age of 7 weeks old, weighing about 200 g, were supplied by Medicinal Laboratory Animal Center of Zhengzhou University, Zhengzhou, China. The rats were kept in individual cages under a 12 h light/dark cycle in an approved animal house facility at Henan Agricultural University, Zhengzhou, China. The rats had free access to standard lab chow and water. This study was carried out in strict accordance with the recommendations in the Guide for the Care and Use of Laboratory Animals of the National Institutes of Health. All experimental protocols were approved by the Institutional Animal Ethics Committee of Henan Agricultural University (Permit Number: 11-0085). All surgery was performed under ether anesthesia, and all efforts were made to minimize suffering.

### Animal model and grouping

Hyperlipidemic rats were induced by high-lipid diet for 4 weeks. The high-lipid diet was prepared as reported [22]–[24] according to the recipe: 1% cholesterol, 0.1% pig bile salt, 10% lard, 5% yolk powder, 5% whole milk powder, 78.9% basal diet (standard lab chow). Standard lab chow for rats was commercially obtained from Medicinal Laboratory Animal Center of Zhengzhou University, Zhengzhou, China. It comprised of 22.5% protein, 4.2% lipid, and 62.3% carbohydrate.

Normally, the rats with high serum lipid levels (TC>15 mmol/L, and TG>1.2 mmol/L) were identified as hyperlipidemic rats [25]. To identify induction of hyperlipidemia, at the end of 3 and 4 week after feeding high-lipid diet, blood sample was collected from tail vein of the rats, and then assayed for serum TC and TG levels using a standard enzymatic assay kit (BioSino Bio-technology and Science Inc., China). After serum lipid determination, 20 rats which were fed with high-lipid diet for 4 weeks all showed hyperlipidemic symptom.

Alfalfa saponin extract (ASE) was provided by Hebei Bao'en Biotechnology Co., Ltd (Shijiazhuang, China), which was extracted from the leaves and stem of alfalfa, and the purity was 62%. The preparation of ASE was as follows: The powdered dried stems and leaves of alfalfa were defatted by soxhlet extraction with petroleum ether (2×24 h), and the defatted power was extracted with 75% ethanol (10 ml/g) for 3 h with constant stirring. After suction filtration, the extraction was repeated. The extracts were combined and evaporated under vacuum. The dried extracts was dissolved in distilled water at a concentration of 100 g/l and then fractionated on a macroporous adsorption resin AB-8 column with distilled water, 50% ethanol respectively. The ethanol extracted saponins were obtained from the 50% ethanol fractions, and evaporated under vacuum.

To evaluate effects of ASE on hyperlipidemic rats, 10 identified hyperlipidemic rats (oral administration with 2 ml distilled water at 9:00 am every morning during the period of hyperlipidemic model establishment) were continuously fed with a high-lipid diet, and at the same time once every morning treated with 240 mg/kg/day ASE in 2 ml distilled water by oral gavage from the beginning of 5 week. The treatments lasted for 4 weeks. Furthermore, to investigate prevention effects of ASE on hyperlipidemic rats, from the beginning of hyperlipidemic model establishment, 10 rats were fed with a high-lipid diet, and at the same time once every morning treated with 240 mg/kg/day ASE in 2 ml distilled water by oral gavage for 8 weeks. The dose of 240 mg/kg was decided based on an earlier study [21]. Hyperlipidemic rats were fed with high-lipid diet and orally administrated with 2 ml distilled water at the same time once every morning for 8 weeks. The control rats were fed with standard lab chow and orally administrated with 2 ml distilled water at the same time once every morning for 8 weeks. So there are 4 groups with 10 rats in each group: control group, hyperlipidemic group, ASE treatment group and ASE prevention group.

### Sampling

#### Data collection

The rats were monitored daily for general health and weighed individually at the beginning and end of the experiment. The daily feed intake and weight gain were recorded during the experimental period.

#### Blood sampling

At the end of 8 week, rats were fasted overnight and killed under ether anesthesia. Blood was collected by cardiac puncture and left at room temperature for coagulation. The serum was obtained by centrifugation at 3000×g, 4°C for 10 min and stored at -70°C for later use. The liver was removed and washed with normal saline, blotted dry on filter paper, weighed, then immediately frozen in liquid nitrogen and stored at −70°C for further analysis.

#### Liver sampling

0.5 g liver of each rat was homogenized in Phosphate Buffered Saline (PBS, pH 7.2) (0.25 g/ml) at 4°C. The supernatant was then centrifuged at 4000×g, 4°C for 10 min. The preparation was adjusted to indicated concentration and stored at −70°C for future use. Protein concentration in supernatant was measured by Bradford method [26].

#### Feces sampling

Feces of each rat were collected during the last 3 days experimental period and dried at 60°C. Feces were weighed and grinded into 0.5 µm diameter powder. 0.5 g Feces powder of each rat were extracted 3 times with 10 ml of 95% ethanol at 60°C and then filtered as well as evacuated thoroughly. The residue was dissolved in PBS by sonication. The preparation was adjusted to indicated concentration and stored at −70°C for later analysis.

### Bioassays

#### Biochemical assay of serum lipid, cholesterol and bile acids in the liver and feces

Levels of triglyceride (TG), total cholesterol (TC), high-density lipoprotein cholesterol (HDL-C) and low-density lipoprotein cholesterol (LDL-C) in serum were determined using the Hitachi 911 analyzer (Roche) with commercial kits (BioSino Bio-technology and Science Inc.) according to the manufacturer's instruction.

Levels of total cholesterol (TC) and total bile acids (TBA) in the rat liver or fecal preparation were determined using the Hitachi 911 analyzer (Roche) with commercial kits (BioSino Bio-technology and Science Inc.) according to the manufacturer's instruction.

#### Enzymatic activity assay

Enzymatic activity of HMGCR, ACAT2 and CYP7A1 as well as concentration of LDLR in rat liver were determined respectively using Rat ELISA Kits (GENMED) [27], [28] according to the manufacturer's instruction using Berthold LB940 microplate reader (Berthold Technologies).

#### RNA preparation, cDNA synthesis and real-time RT-PCR

Total RNA was prepared using Trizol reagent (Invitrogen) according to the manufacturer's protocol. One microgram RNA was transcribed into cDNA using Omniscript reverse transcriptase (QIAGEN) according to manufacturer's protocol. Real-time quantitative PCR (QPCR) was used to detect the expression difference of *Hmgcr*, *Acat2, Cyp7a1* and *Ldlr* in hepatic tissues among treatments. *β-Actin* was used as an internal control. The primers for QPCR were presented in Table 1. Real-time QPCR was performed on a 96-well PCR plate in triplicate with a total reaction volume of 10 µL containing 1 µL cDNA, 5 uL SYBRGreen Mastermix, 0.1 uL of each specific forward and reverse primers, and 3.8 µL nuclease-free water, PCR was carried out in an ABI PRISM 7700 sequence detection system (Applied Biosystems) with 2 min at 95°C for predegeneration, then followed by 40 cycles at 95°C for 15 s, 60°C for 20 s and 72°C for 30 s each. The reaction mixture with no cDNA was considered as the negative control to confirm the absence of primer dimerization. The cycle threshold (Ct) values were determined by Sequence Detection System software version 1.7a. Qualitative PCR was performed to confirm formation of a single product in each reaction before quantitation. The target gene expressions of the samples were exhibited as fold change from control. All genes were normalised with *β-Actin*.

**Table 1:** SYBR Green primer sequences used for real-time RT-PCR.

Gene	Accession No.	Forward primer *(5′→ 3′)*	Reverse primer *(5′→ 3′)*	Size (bp)
*β-Actin*	NM_031144	GGGACCTGACAGACTACCTC	AAGTCTAGGGCAACATAGCAC	121
*Hmgcr*	NM_013134.2	TGTCATTCCAGCCAAGGTG	ATGGGCGTTGTAGCCTCCT	127
*Acat2*	AB075946.1	CTCTGCTGCTGTCCATCC	CCAGGTGCGGTAATAGTTG	179
*Cyp7a1*	J05430.1	CAGGGAGATGCTCTGTGTTCA	AGGCATACATCCCTTCCGTGA	121
*Ldlr*	NM_175762.2	TGTGGGTTCCATAGGGTT	CTGGTCCATCACGGCGC	193

*Hmgcr*, 3-Hydroxy-3-methylglutaryl CoA reductase; *Acat2*, acyl-CoA: cholesterol O-acyltransferase 2; *Cyp7a1*, cytochrome P450, family 7, subfamily a, polypeptide 1; *Ldlr*, low-density lipoprotein receptor.

### Statistical analysis

All results were expressed as mean±SEM. The data were evaluated by one-way ANOVA, and the differences between the means were assessed using Duncan's test. *P*<0.05 was considered as statistically significant.

## Results

### Effect of ASE on body weight gain and relative liver weight of rats

**Figure 1:**
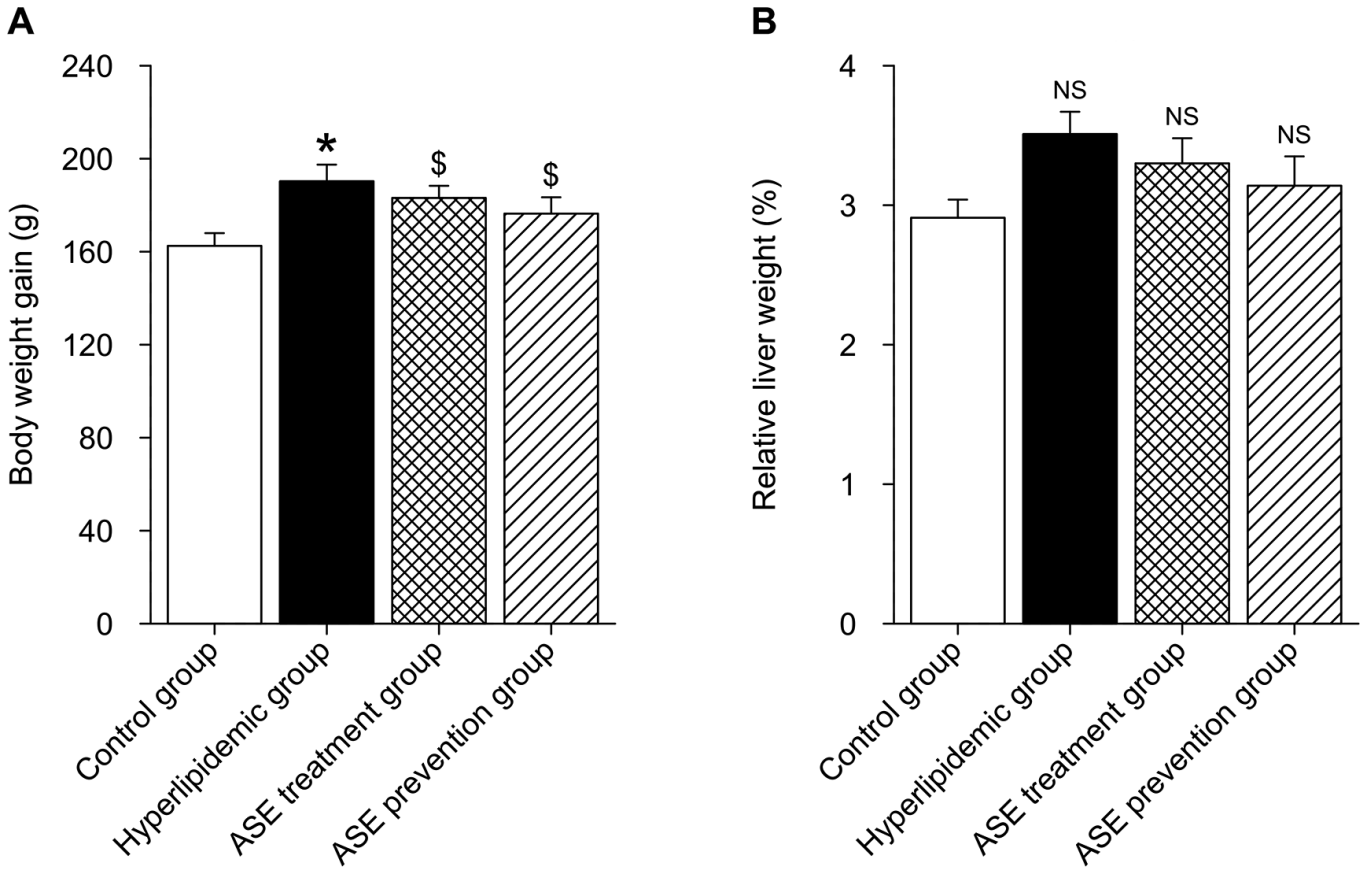
Effects of alfalfa saponin extract on body weight gain and relative liver weight of rats. A. Body weight gain. B. Relative liver weight. n = 10. * *P*<0.05, Hyperlipidemic group *VS.* control group. $ *P*<0.05, ASE group (both ASE treatment and prevention group) *VS.* control group. NS, not significant (*P*>0.05).

Data of body weight gain and relative liver weight of rats were shown in Figure 1. The results indicated that the intake of high-lipid diet significantly increased body weight gain of rats compared with the control group (*P*<0.05). Although there were no significant differences in body weight gain between ASE administration group and hyperlipidemic group (*P* = 0.084 hyperlipidemic group *vs* ASE treatment group; *P* = 0.067 hyperlipidemic group *vs* ASE prevention group), but the administration of ASE had a trend to reduce body weight gain of hyperlipidemic rats although they did not reach the values of control group (*p*<0.05 control *vs* both ASE administration groups). No significant differences in relative liver weight of rats among treatments were observed (*P* = 0.163).

### Effects of ASE on serum lipid levels of rats

**Figure 2:**
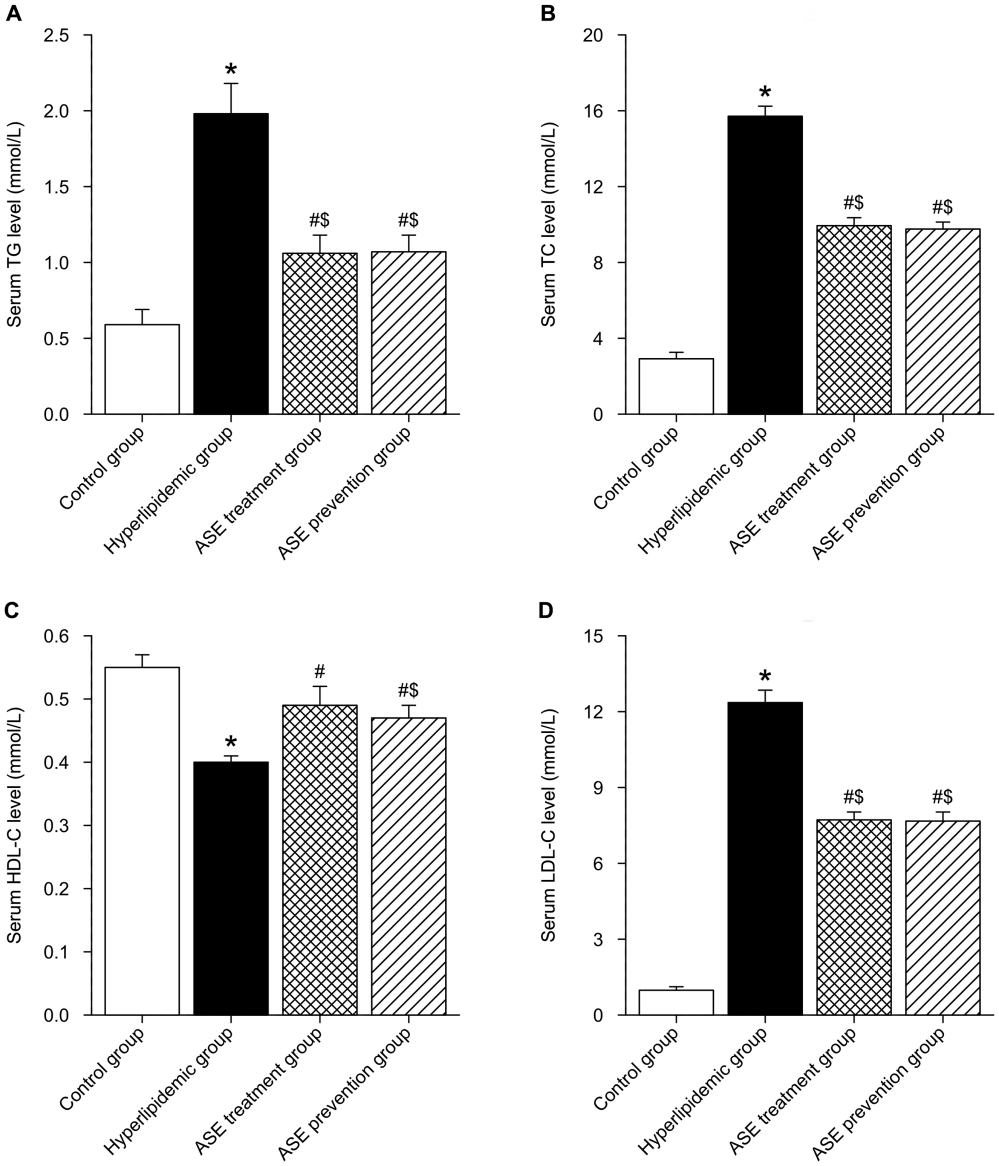
Effects of alfalfa saponin extract on serum lipid levels of rats. A. Serum TG level. B. Serum TC level. C. Serum HDL-C level. D. Serum LDL-C level. n = 10. TG, triglycerides; TC, total cholesterol; HDL-C, high-density lipoprotein cholesterol; LDL-C, low-density lipoprotein cholesterol; * *P*<0.05, Hyperlipidemic group *VS.* control group; # *P*<0.05, ASE group (both ASE treatment and prevention group) *VS.* hyperlipidemic group; $ *P*<0.05, ASE group (both ASE treatment and prevention group) *VS.* control group.

As summarized in Figure 2, serum TG, TC and LDL-C levels were markedly elevated (*P*<0.05), whereas serum HDL-C levels were significantly decreased in hyperlipidemic rats compared with the control group (*P*<0.05). Administration of ASE (both ASE prevention group and treatment group) led to significant reduction of serum TG, TC and LDL-C levels (*P*<0.05), as well as the rise of serum HDL-C levels compared to hyperlipidemic group (*P*<0.05) although they did not reach the values of control group (*p*<0.05 control *vs* both ASE administration groups), which indicated the beneficial effects of ASE on serum lipid profiles in hyperlipidemic rats.

### Effects of ASE on total cholesterol and total bile acids levels in liver and feces of rats

**Figure 3:**
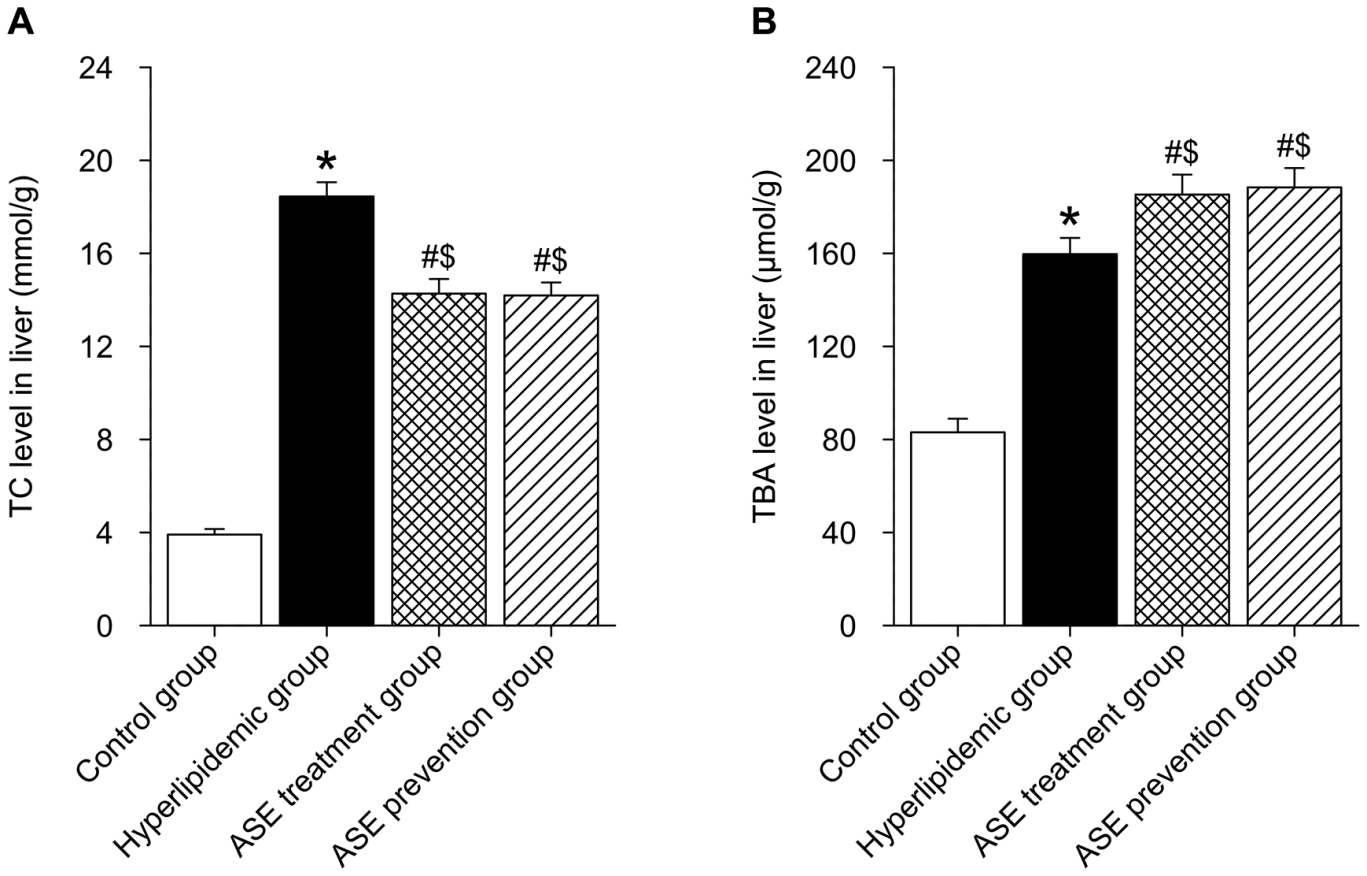
Effects of alfalfa saponin extract on total cholesterol and total bile acids levels in liver of rats. A. TC level in liver. B. TBA level in liver. n = 10. TC, total cholesterol; TBA, total bile acids; * *P*<0.05, Hyperlipidemic group *VS.* control group; # *P*<0.05, ASE group (both ASE treatment and prevention group) *VS.* hyperlipidemic group; $ *P*<0.05, ASE group (both ASE treatment and prevention group) *VS.* control group.

**Figure 4:**
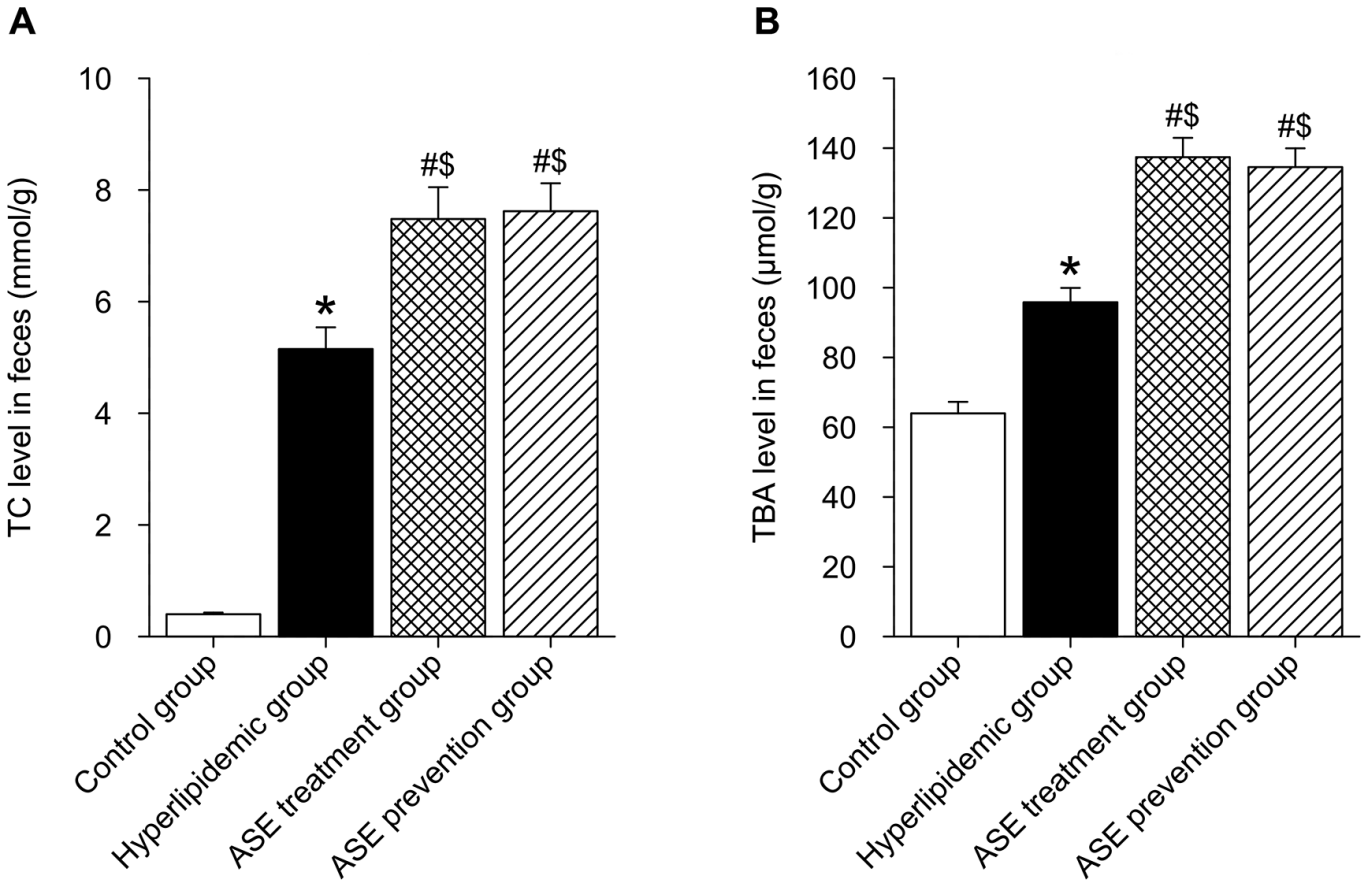
Effects of alfalfa saponin extract on total cholesterol and total bile acids levels in feces of rats. A. TC level in feces. B. TBA level in feces. n = 10. TC, total cholesterol; TBA, total bile acids; * *P*<0.05, Hyperlipidemic group *VS.* control group; # *P*<0.05, ASE group (both ASE treatment and prevention group) *VS.* hyperlipidemic group; $ *P*<0.05, ASE group (both ASE treatment and prevention group) *VS.* control group.

As summarized in Figure 3 and 4, the rats fed with high-lipid diet showed markedly higher levels of liver TC and TBA compared with the control group (*P*<0.05), ASE administration (both ASE prevention group and treatment group) significantly reduced liver TC level (*P*<0.05) although they did not reach the values of control group (*p*<0.05 control *vs* both ASE administration groups), however both ASE administration significantly increased liver TBA level (*P*<0.05). Both TC and TBA levels in feces of rats fed with high-lipid diet were significantly higher than those in control rats (*P*<0.05), and further remarkably elevated by both ASE administration (*P*<0.05).

### Effects of ASE on gene expression and enzymatic activity in liver of rats

**Figure 5:**
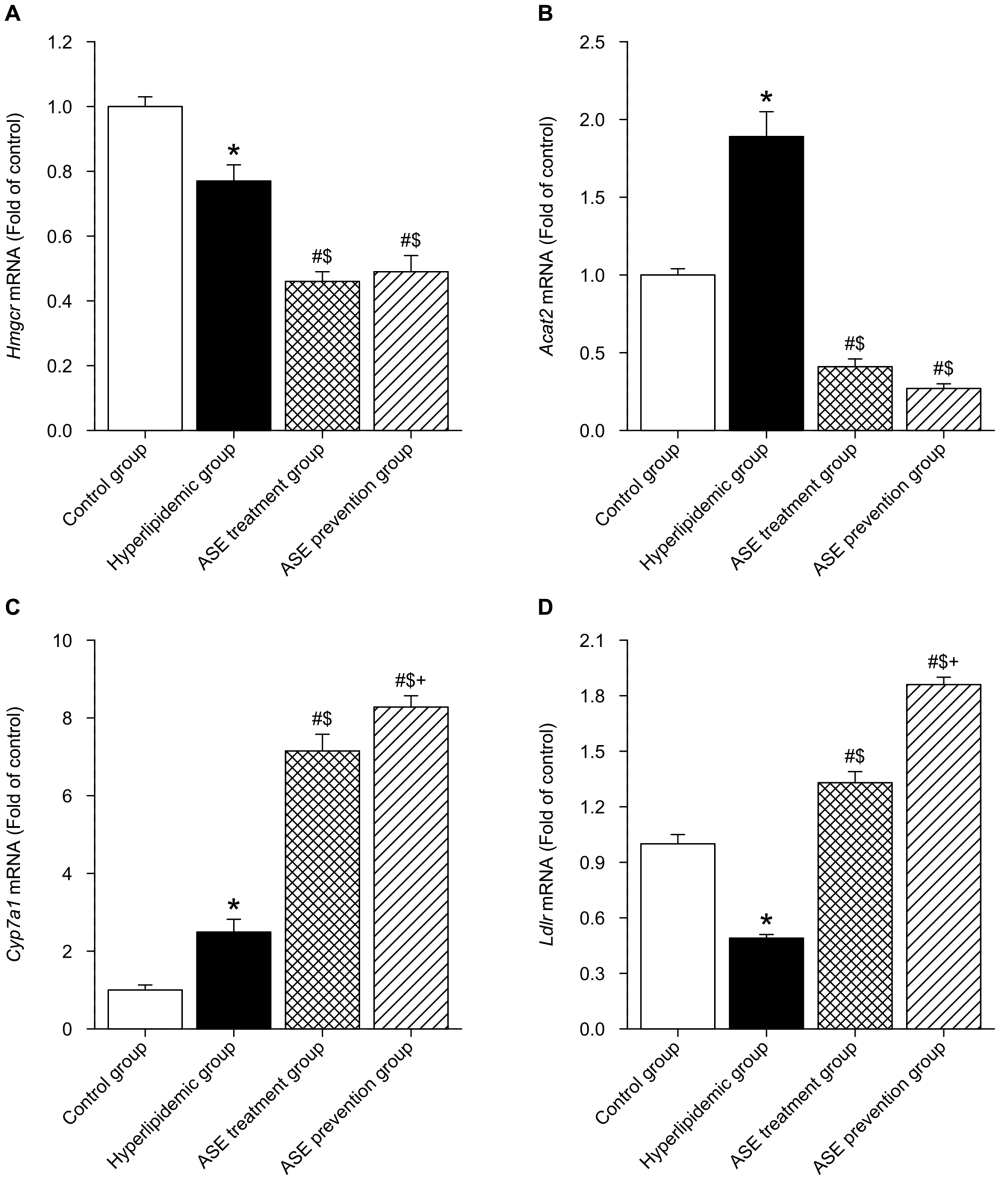
Effects of alfalfa saponin extract on mRNA expression of genes in rat liver. A. *Hmgcr* mRNA. B. *Acat2* mRNA. C. *Cyp7a1* mRNA. D. *Ldlr* mRNA. n = 10. *Hmgcr*, 3-Hydroxy-3-methylglutaryl CoA reductase; *Acat2*, acyl-CoA: cholesterol O-acyltransferase 2; *Cyp7a1*, cytochrome P450, family 7, subfamily a, polypeptide 1; *Ldlr*, low-density lipoprotein receptor. * *P*<0.05, Hyperlipidemic group *VS.* control group; # *P*<0.05, ASE group (both ASE treatment and prevention group) *VS.* hyperlipidemic group; $ *P*<0.05, ASE group (both ASE treatment and prevention group) *VS.* control group; + *P*<0.05, ASE prevention group *VS.* ASE treatment group.

**Figure 6:**
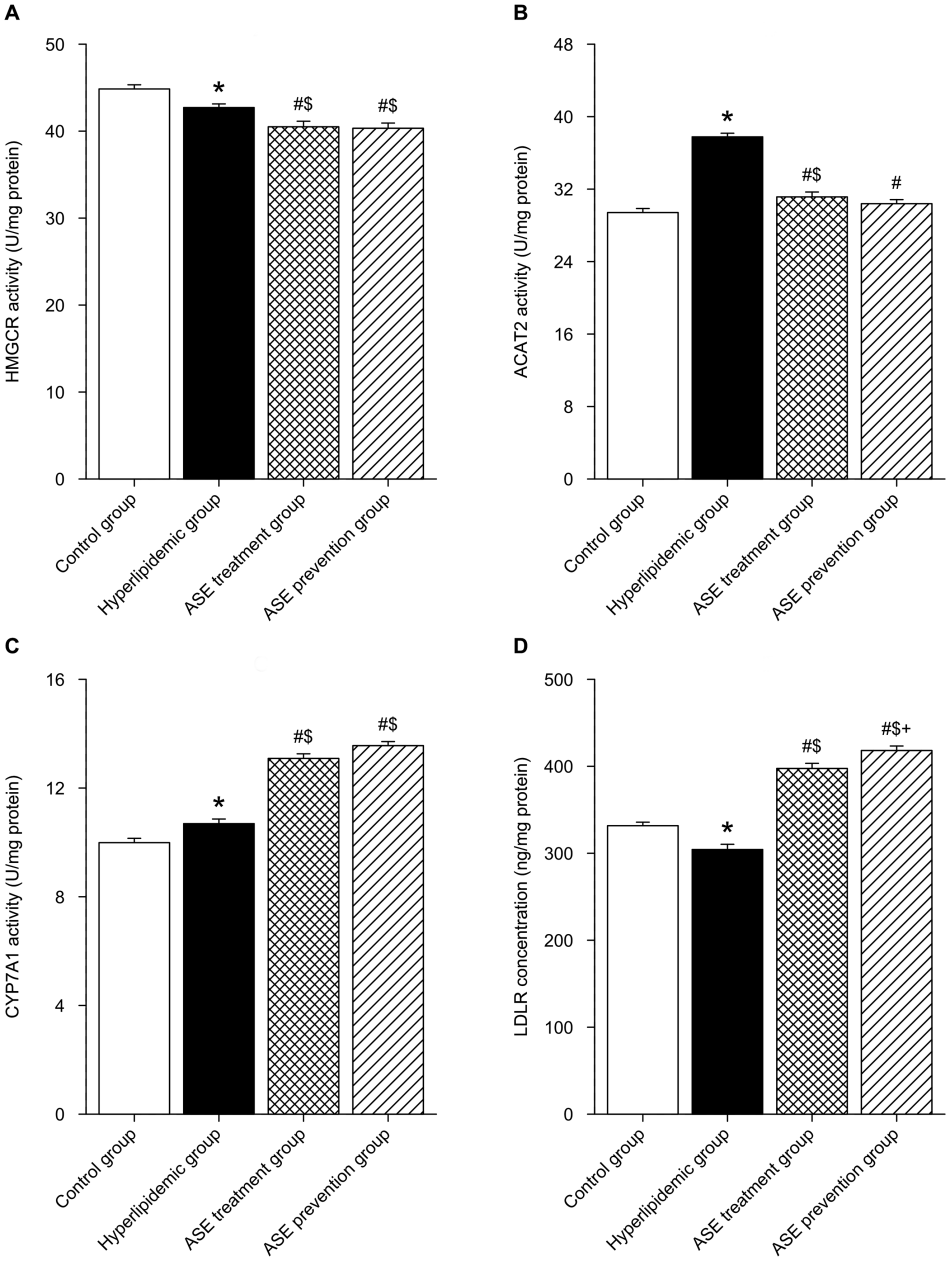
Effects of alfalfa saponin extract on enzymatic activity and concentration of LDLR in rat liver. A. HMGCR activity. B. ACAT2 activity. C. CYP7A1 activity. D. LDLR concentration. n = 10. HMGCR, 3-Hydroxy-3-methylglutaryl CoA reductase; ACAT2, acyl-CoA: cholesterol O-acyltransferase 2; CYP7A1, cytochrome P450, family 7, subfamily a, polypeptide 1; LDLR, low-density lipoprotein receptor. * *P*<0.05, Hyperlipidemic group *VS.* control group; # *P*<0.05, ASE group (both ASE treatment and prevention group) *VS.* hyperlipidemic group; $ *P*<0.05, ASE group (both ASE treatment and prevention group) *VS.* control group; + *P*<0.05, ASE prevention group *VS.* ASE treatment group.

RT-PCR data presented in Figure 5 showed that gene expression of *Hmgcr* in liver of hyperlipidemic rats was down-regulated as compared with the control group (*P*<0.05), and further markedly reduced in hyperlipidemic rats with ASE administration (both ASE prevention group and treatment group) (*P*<0.05). On the contrary, gene expression of *Cyp7a1* in liver of hyperlipidemic rats was up-regulated as compared with the control group (*P*<0.05), and further dramatically elevated in hyperlipidemic rats with both ASE administration (*P*<0.05). Gene expression of *Acat2* in liver of hyperlipidemic rats was up-regulated as compared to the control group (*P*<0.05), and remarkably decreased in hyperlipidemic rats with both ASE administration (*P*<0.05), even they were lower than the values of control group (*p*<0.05 control *vs* both ASE administration groups). On the contrary, gene expression of *Ldlr* in liver of hyperlipidemic rats was down-regulated as compared to the control group (*P*<0.05), and significantly increased in hyperlipidemic rats with both ASE administration (*P*<0.05) although they did not reach the values of control group (*p*<0.05 control *vs* both ASE administration groups). Compared with ASE treatment group, gene expression of *Cyp7a1* and *Ldlr* of rats in ASE prevention group were significantly increased (*P*<0.05). ELISA data presented in Figure 6 showed that there was the same trend on activities of these enzymes in the liver as gene expression.

## Discussion

### Anti-hyperlipidemic effects of ASE in hyperlipidemic rats

Dyslipidemia (usually elevated serum levels of TG, TC and LDL-C, accompanied by reduced HDL-C level) is a metabolic disorder that constitutes a crucial risk factor of atherosclerosis and cardiovascular disease [29]. LDL-C has been found to be the most dangerous factor among serum lipids owing to increased penetration of oxidated LDL-C into arterial walls [30] and then the excess of LDL is easily deposited into the blood vessel walls, which is involved in the initiation and promotion of atherosclerosis and becomes a major component to cause atherosclerotic plaque lesions [31]. HDL carries cholesterol and cholesterol esters from the peripheral tissues and cells to the liver, where cholesterol is metabolized into bile acids. So HDL plays a very important role to reduce cholesterol levels in the blood and peripheral tissues, and to inhibit atherosclerotic plaque formation in the aorta [32]. Therefore, decreasing serum TC and LDL-C levels and increasing serum HDL level are pivotal for reducing the risk of atherosclerosis [33].

Diet plays a crucial role in the control of cholesterol homeostasis. The consumption of cholesterol-enriched diet is regarded as a key risk factor in the development of cardiovascular diseases as it leads to the development of hyperlipidemia and atherosclerosis. Our results showed that the hyperlipidemic rats developed higher serum levels of TG, TC and LDL-C, as well as a decreased concentration of HDL-C. The results obtained were consistent with the previous studies [20], [21], [34], [35]. However, the elevated serum levels of TG, TC and LDL-C in hyperlipidemic rats were significantly reduced by ASE administration. Conversely, the declined serum HDL-C level was significantly increased by ASE administration. These results suggested that ASE was an effective lipid-lowering agent. The findings also agreed with the previous studies on the cholesterol-lowering effects of AS in monkeys and rats reported by Malinow et al [10]–[12] and Story et al [14], they concluded that the hypocholesterolemic effects of AS could be ascribed to its inhibition of cholesterol absorption. However, whether the cholesterol-lowering effects of ASE are mediated by some key genes involved in cholesterol metabolism is not known. Therefore, in the present study, we investigated the hepatic metabolic pathway of cholesterol and detected the expression and activity of HMGCR, ACAT2, CYP7A1 and LDLR in the liver.

### Inhibitory effects of ASE on HMGCR and ACAT2 in hyperlipidemic rats

3-Hydroxy-3-methylglutaryl CoA reductase (HMGCR) is the rate-limiting enzyme in cholesterol biosynthesis. The inhibition of HMGCR expression or activity will lead to inhibit cholesterol de novo synthesis in the liver and thus reduce serum cholesterol level [16], [36]. Acyl-CoA: cholesterol O-acyltransferase 2 (ACAT2), is the major tissue cholesterol-esterifying enzyme, which is found within lipoprotein-producing cells such as enterocytes and hepatocytes [18]. It has been previously documented that ACAT2 converts free cholesterol into cholesteryl esters in response to excess intracellular cholesterol [37]. ACAT2-derived cholesteryl esters may also be incorporated into hepatic apoB-containing lipoproteins and secreted into plasma. So ACAT2 plays a critical role in the production of atherogenic apoB-containing lipoproteins and that ACAT2-specific inhibitors are extremely effective in preventing murine atherosclerosis [38].

In our study, gene expression of *Hmgcr* was suppressed in hyperlipidemic rats, and further significantly inhibited by ASE administration, and markedly reduced TC level in the liver and serum of hyperlipidemic rats. So the decreasing TC level in the liver and serum observed in the current study could be explained by the down-regulation of *Hmgcr*, which reduced the conversion of HMG-CoA into mevalonate, and inhibited the synthesis of cholesterol [39]. Although there was no report on the effect of ASE on the expression of cholesterol metabolism related genes, it was found that FTZ (*Fufang Zhenshu Tiao Zhi*) extracted from Chinese herbs could regulate the gene expression of *Hmgcr* [28].

Cynomolgus monkeys fed a high-cholesterol diet express increased hepatic *Acat2* mRNA levels, and patients treated with statins have a dose-dependent decrease in *Acat2* expression [40]. The intake of high-lipid diet led to an increase in *Acat2* mRNA levels in the present study, however, the administration of ASE remarkably decreased gene expression of *Acat2*. Studies of Alger et al [41] demonstrated that liver-specific depletion of *Acat2* with antisense oligonucleotides prevented dietary cholesterol-associated hepatic steatosis both in an inbred mouse model of non-alcoholic fatty liver disease (SJL/J) and in a humanized hyperlipidemic mouse model (LDLr^−/−^, apoB^100/100^), and ACAT2-specific inhibitors might hold unexpected therapeutic potential to treat both athero-sclerosisand non-alcoholic fatty liver disease. So the down-regulation of *Acat2* in the current study might prevent dietary cholesterol-associated hepatic steatosis. Further study on the effect of ASE on the gene expression of *Acat2* could give more insights on the pharmacological effects of AS.

### Up-regulating effects of ASE on CYP7A1 and LDLR in hyperlipidemic rats

Cholesterol conversion into bile acids in the liver is a pivotal pathway in reducing the serum cholesterol level. Bile acid synthesis and excretion contribute to most of the cholesterol removed from the body [42]. Cytochrome P450, family 7, subfamily a, polypeptide 1, also known as cholesterol 7-alpha-hydroxylase (CYP7A1) is the rate-limiting enzyme in the classical bile acid biosynthetic pathway, which accounts for at least 75% of the total bile acid pool [43]. The increase of CYP7A1 expression or activity will enhance the catabolic pathway of cholesterol and led to the reduction of serum and liver cholesterol level [44]. Low-density lipoprotein receptor (LDLR) is a cell surface glycoprotein, which binds two proteins: apoB-100, which is the sole protein of LDL, and apoE, which is found in multiply copies in IDL and a subclass of HDL [19]. LDLR has dual role in LDL metabolism. First, it limits LDL production by enhancing the remove of the precursor, IDL, from the circulation. Second, it enhances LDL degradation by mediating cellular uptake of LDL. A deficiency of LDL receptors causes LDL to accumulate as a result both of overproduction and of delayed removal [45]. So LDLR is a crucially important modulator of plasma LDL levels in humans and animals. The increase of LDLR expression or activity will result in the reduction of serum LDL cholesterol level by enhancing the uptake and clearance of LDL cholesterol [46].

Our study showed that gene expression of *Cyp7a1* was enhanced in liver of hyperlipidemic rats and further significantly increased by ASE administration, which resulted in an increase of the cholesterol conversion into bile acids. Gene expression of *Ldlr* was inhibited in hyperlipidemic rats, however, ASE promoted hepatic uptake and clearance of plasma cholesterol by up-regulating gene expression of *Ldlr*. The liver plays an important role in maintaining whole-body cholesterol homeostasis. It is the major site for elimination of cholesterol from the body via bile through converting cholesterol into bile acids, and also a major catabolic site for the LDL receptor-mediated pathway [47], [48]. In general, the rise in expression of *Cyp7a1* and *Ldlr* would increase uptake of LDL cholesterol and enhance the catabolic pathway which converts cholesterol to bile acid. As a result, hepatic TBA level would increase, hepatic and serum TC level would reduce. This corresponded to the changes in these parameters observed in the present study. Reena et al. [46] reported the hypocholesterolemic effects of interesterified oils were mediated by up-regulating *Cyp7a1* and *Ldlr* mRNA expression in rats. Wu et al. [49] also reported PNS (*Panax notoginseng* saponins) supplementation could up-regulate the mRNA expression of *Cyp7a1* and supress the diet-induced hypercholesterolaemia. Similar mechanism was also observed with soyisoflavone and puerarin which decreased serum TC level by mainly enhancing the expression of *Cyp7a1* [50], [51].

Cholesterol homeostasis is tightly controlled by coordinated changes in the concentrations of mRNA encoding multiple enzymes [15]. The activity of these enzymes in the present study also paralleled the observed changes in mRNA levels. Down-regulating gene expression of *Hmgcr* and *Acat2* thus decreasing their activity by ASE administration may have resulted in the reduction of liver and serum TC level in hyperlipidemic rats. Conversely, up-regulating gene expression of *Cyp7a1* and *Ldlr* thus increasing their activity by ASE administration may have led to the rise of TBA level and the reduction of TC level in the liver and serum of hyperlipidemic rats. The data in our study suggested ASE could regulate cholesterol metabolism mainly from three pathways, i. e. enhancing the catabolic pathway and uptake of LDL-C and inhibiting the synthesis pathway of cholesterol. The hypocholesterolemic effects of some plant extracts was also found to be related to the expression of genes implicated in cholesterol metabolism, such as *Hmgcr* and *Cyp7a1* in FTZ [28], *Cyp7a1* in PNS [49], soyisoflavone [50] and puerarin [51]. Although the exact mechanism of action for these plant extracts on cholesterol metabolism remains to be elucidated, the results obtained provide powerful support for seeking new natural cholesterol-lowering agents.

Our study also showed that levels of TBA and TC in feces of hyperlipidemic rats were significantly increased by ASE administration, which also suggested that the increase in excretion of cholesterol and its metabolite was another important pathway of ASE to reduce serum cholesterol level in hyperlipidemic rats. Previously, it has been demonstrated that AS reduced serum and/or liver cholesterol accumulation, But these studies mainly focused on the effects of AS on intestinal cholesterol adsorption, which indicated AS bind to cholesterol preventing its reabsorption, increasing the cholesterol content in the feces and decreasing cholesterol levels in the blood [10]–[14]. In the present study, besides the adsorption of ASE on cholesterol, it was notable that our findings demonstrated that the regulation of ASE on some key genes implicated in cholesterol metabolism might be responsible for the hypocholesterolemic effects of ASE. However, whether ASE has a direct effect in mRNA expression and activity of these genes, further study is needed to clarify the detailed mechanism.

Although most parameters evaluated didn't return to normal values in both ASE administration groups compared with the control group, but these parameters were significantly improved in both ASE administration groups compared with hyperlipidemic group, which suggested that oral administration of ASE was able to ameliorate plasma and liver cholesterol/lipid parameters. These changes were correlated with alterations in the mRNA expression and activity of key genes implicated in cholesterol metabolism. There was no significant difference between ASE treatment and ASE prevention group for most parameters evaluated, which indicated that ASE prevention group had no extra effects in comparison with ASE treatment group, so further experiments should be conducted to investigate the preventive effects of ASE on hyperlipidemia.

## Conclusions

Our present study indicated that ASE had cholesterol-lowering effects. The possible mechanism could be attributed to: (1) the down-regulation of *Hmgcr* and *Acat2*, as well as up-regulation of *Cyp7a1* and *Ldlr* in liver of hyperlipidemic rats, which involved in cholesterol biosynthesis, uptake, and efflux pathway; (2) the increase in excretion of cholesterol. The findings in our study suggested ASE had great potential usefulness as a natural agent for treating hyperlipidemia.
